# Electrochemically Enhanced Drug Delivery Using Polypyrrole Films

**DOI:** 10.3390/ma11071123

**Published:** 2018-07-01

**Authors:** Sayed Ashfaq Ali Shah, Melike Firlak, Stuart Ryan Berrow, Nathan Ross Halcovitch, Sara Jane Baldock, Bakhtiar Muhammad Yousafzai, Rania M. Hathout, John George Hardy

**Affiliations:** 1 Department of Chemistry, Lancaster University, Lancaster, LA1 4YB, UK; ashfaqali66@yahoo.com (S.A.A.S.); m.firlak@gmail.com (M.F.); Stuart_Berrow@yahoo.co.uk (S.R.B.); n.r.halcovitch@lancaster.ac.uk (N.R.H.); s.baldock@lancaster.ac.uk (S.J.B.); rania.hathout@pharma.asu.edu.eg (R.M.H.); 2 Department of Chemistry, Government Post Graduate College No. 1, Abbottabad 22010, Pakistan; 3 Department of Chemistry, Hazara University, Mansehra 21130, Pakistan; yousafzaibm@gmail.com; 4 Department of Pharmaceutics and Industrial Pharmacy, Faculty of Pharmacy, Ain Shams University, Cairo 11566, Egypt; 5 Bioinformatics Program, Faculty of Computer and Information Sciences, Ain Shams University, Cairo 11566, Egypt; 6 Department of Pharmaceutical Technology, Faculty of Pharmacy and Biotechnology, German University in Cairo, Cairo 11835, Egypt; 7 Materials Science Institute, Lancaster University, Lancaster, LA1 4YB, UK

**Keywords:** conducting polymers, electroactive polymers, medical devices, drug delivery, anti-inflammatory, antibiotic

## Abstract

The delivery of drugs in a controllable fashion is a topic of intense research activity in both academia and industry because of its impact in healthcare. Implantable electronic interfaces for the body have great potential for positive economic, health, and societal impacts; however, the implantation of such interfaces results in inflammatory responses due to a mechanical mismatch between the inorganic substrate and soft tissue, and also results in the potential for microbial infection during complex surgical procedures. Here, we report the use of conducting polypyrrole (PPY)-based coatings loaded with clinically relevant drugs (either an anti-inflammatory, dexamethasone phosphate (DMP), or an antibiotic, meropenem (MER)). The films were characterized and were shown to enhance the delivery of the drugs upon the application of an electrochemical stimulus in vitro, by circa (ca.) 10–30% relative to the passive release from non-stimulated samples. Interestingly, the loading and release of the drugs was correlated with the physical descriptors of the drugs. In the long term, such materials have the potential for application to the surfaces of medical devices to diminish adverse reactions to their implantation in vivo.

## 1. Introduction

The market for global drug-delivery technologies is a multibillion-dollar industry, and there is a growing demand for drug-delivery devices in both developed and emerging economies (in part, driven by aging societies and rapid urbanization) [1,2]. The market for implantable medical devices is also a multibillion-dollar industry, with a similarly growing demand driven by the same factors (i.e., increasing geriatric populations and incidences of chronic diseases, coupled with the adoption of implantable medical devices) [3,4,5]. Medical devices are implanted in either hard tissues (e.g., orthopedic implants such as reconstructive joint replacements, dental implants, and spinal implants) or soft tissues (e.g., intraocular lenses, or the skin). The successful integration of such devices is dependent on the availability of sterile surgical conditions, patient health, etc., and their implantation is most commonly coupled with a course of condition- and patient-specific drugs [6,7].

Drug-delivery systems can be engineered to deliver drugs at rates controlled by specific features of the systems, particularly their chemical composition (e.g., inorganic/organic components, molecular weights of their constituents, crosslinking density of polymers, etc.) and the inclusion of components that respond to chemical stimuli (e.g., enzymes, ions, or pH) or physical stimuli (e.g., electromagnetic fields, or temperature) [8,9,10,11,12,13,14,15].

Responsiveness to electric fields is an inherent property of electrically conducting materials (e.g., metals), and certain molecules respond to the application of electric fields by orienting their dipoles with the applied field, whereas other molecules can undergo redox reactions in response to electricity. An exciting class of electrically conducting materials is that of organic electronic materials (OEMs). Various types of OEMs exist, including fullerenes (bucky balls or nanotubes), graphene/graphene oxide, or conjugated polymers (e.g., polyaniline, polypyrrole, or polythiophene). Some OEMs are commercially available, and their properties can be tailored (through chemical modification or the generation of composites) to suit the delivery of various drugs [16,17].

While OEM-based nanoparticles have promise for simultaneous imaging and drug delivery (i.e., theranostic applications) [18,19], nanoparticles are not the only morphology of materials that OEMs can be processed into, and it is also possible to manufacture OEM-based films, fibers, foams, and hydrogels [20,21,22,23,24]. The morphologies of these alternative materials are under investigation for their inclusion into new versions of a variety of clinically translated electronic interfaces for the body (e.g., cardiac pacemakers, cochlear implants, retinal prostheses, and electrodes for deep brain stimulation), or indeed, electronic interfaces for the peripheral nervous system (e.g., for the control of the bladder) [20,21,22,23,24]. The clinically translated examples of electronic interfaces for the body are all currently metal-based (typically connected to batteries implanted at the same time), and the mechanical properties of these metals are markedly different from the soft tissues in which they are implanted (known as a mechanical mismatch). Mechanical mismatches lead to inflammatory responses and the formation of scar tissue around the electronic interface [23,24]. Mismatches can potentially be diminished by coating the surface of the metals with relatively soft OEM-based materials [24], or indeed, the delivery of anti-inflammatories from OEM-based materials [25]. Moreover, it is noteworthy that the surgical procedures necessary to implant such devices are complex, and problems associated with microbial infections in the proximity of these devices can potentially be addressed through the delivery of antimicrobials [16,17,25,26].

Conjugated polymers have fascinating optoelectronic properties, and are consequently being developed for use in the electronic industry [27,28,29,30]. There is academic and industrial interest in their potential application in the biomedical industry for use as bioactuators, biosensors, drug-delivery devices, neural electrode coatings, or indeed, tissue scaffolds for tissue engineering [20,21,22,31]. Polyaniline, polypyrrole, and polythiophene derivatives are most commonly investigated for biomedical applications, and polyaniline- and polypyrrole-based systems were shown to be capable of delivery of a variety of drugs (including anions and, less frequently, cations) [16,17,25,26,32,33,34,35,36].

Prospects for the clinical translation of conjugated polymer-based drug-delivery systems are clearly dependent on their biocompatibility. Histological analyses of tissue in the vicinity of polypyrrole (PPY)-based materials implanted subcutaneously or intramuscularly in rats revealed immune cell infiltration comparable to Food and Drug Administration (FDA) approved poly(lactic-co-glycolic acid) [37], or FDA-approved poly(d,l-lactide-co-glycolide) [38]. Similarly low inflammatory responses were observed for PPY-based materials implanted at the interface of the coronary artery of rats after five weeks [39], or PPY-based sciatic-nerve guidance channels implanted in rats after eight weeks [40], and importantly, PPY-coated electrodes in rat brains after three or six weeks [41]. The implantation of poly(3,4-ethylenedioxythiophene) (PEDOT)-coated electrodes in rat brains resulted in a modest global tissue reaction of approximately the same magnitude as that for silicon probes [42], whereas there was no observable immune response after one week for PEDOT-based materials implanted subcutaneously [43]. The implantation of polyaniline (PANI)-based materials implanted subcutaneously in rats showed low levels of inflammation after four [44], or 50 weeks [45]. We recognize that differences in individual studies (e.g., composition/structure of the materials, animal/tissue models, and the methods used to evaluate immune responses) present challenges when attempting to directly compare the results of each study; however, conjugated polymer-based biomaterials have levels of immunogenicity that are comparable to other FDA-approved biomaterials, and have prospects for clinical translation in the long term.

**Figure 1:**
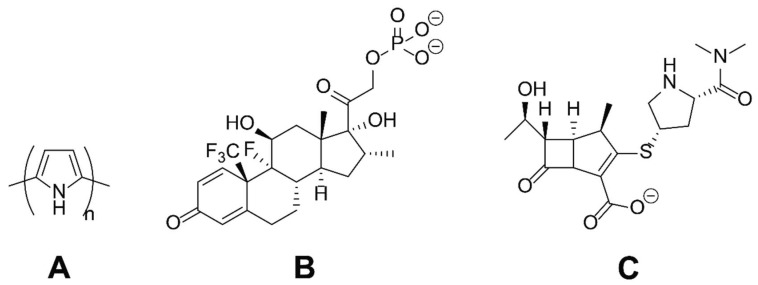
The chemical structures of the substances studied herein: (**A**) Polypyrrole (PPY); (**B**) dexamethasone phosphate (DMP); (**C**) meropenem (MER).

With a view to the long-term development of surface coatings for medical devices (e.g., neural electrodes) to diminish adverse reactions to their implantation in vivo, we report the use of conducting polymer-based coatings that enhance the delivery of drugs upon the application of an electrical potential. As a simple model system, we used polypyrrole (PPY) loaded with clinically relevant drugs (either an anti-inflammatory, dexamethasone phosphate (DMP), or an antibiotic, meropenem (MER)), as depicted in Figure 1. The rationale behind the delivery of DMP was to address problems of local tissue inflammation in the proximity of the materials, whereas the rationale behind the delivery of MER was to help diminish the potential for microbial infections in the proximity of materials that might be associated with the complicated surgical procedures necessary to implant electronic interfaces for the body. The films were characterized using microscopic, spectroscopic, and electrochemical techniques; the delivery of the drugs into a biomedically relevant buffer (phosphate-buffered saline, PBS) was studied in vitro, and the correlation between drug loading/release was correlated with the physical descriptors of the drugs. Such materials have prospects for the preparation of conformal electroactive coatings for implantable biomaterials.

## 2. Results

Films of PPY loaded with an anionic drug (either DMP or MER, see Figure 1) were deposited onto the surface of indium tin oxide (ITO)-coated glass electrodes via electropolymerization. The electrochemical oxidation (at a potential of 1.0 V) and polymerization of pyrrole on the anode (the ITO-coated glass electrode) yielded a film where the positive charges on the backbone of the polypyrrole were counterbalanced by the presence of the anionic dopants from the electrolyte during electropolymerization (in this case one of the anionic drugs, DMP or MER).

**Figure 2:**
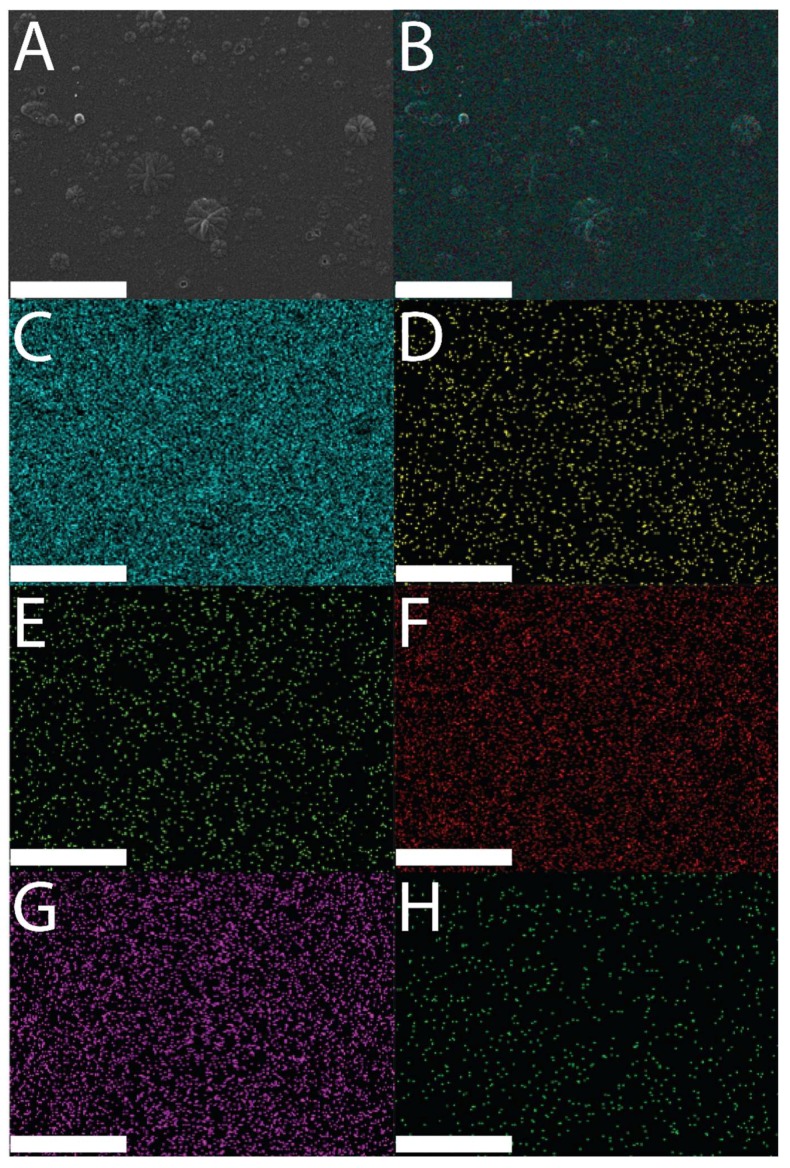
Scanning electron microscopy (SEM) and energy dispersive X-ray spectroscopy (EDX) images of a DMP-doped PPY film: (**A**) SEM image of a DMP-doped PPY film; (**B**) EDX layered image from a DMP-doped PPY film; (**C**) Kα emission of C; (**D**) Kα emission of F; (**E**) Kα emission of N; (**F**) Kα emission of O; (**G**) Kα emission of P; (**H**) Kα emission of Si. Scale bars represent 100 µm.

**Figure 3:**
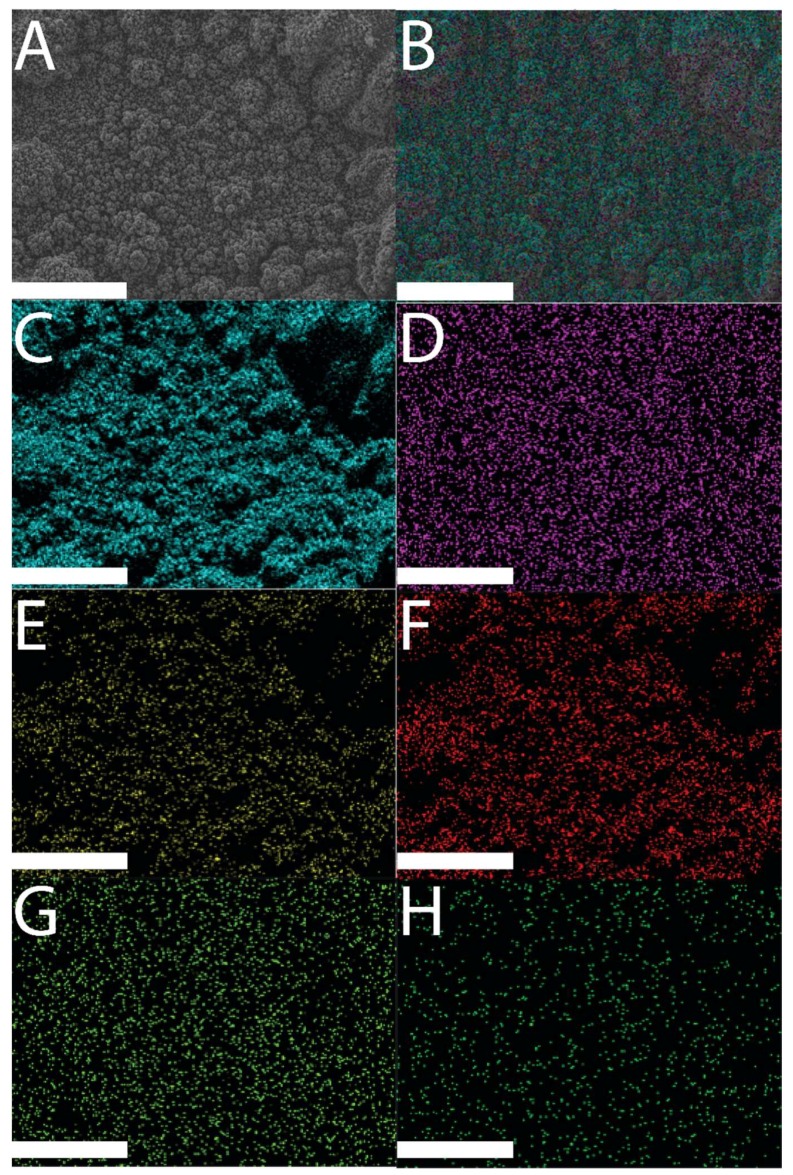
SEM and EDX images of an MER-doped PPY film: (**A**) SEM image of an MER-doped PPY film; (**B**) EDX layered image from an MER-doped PPY film; (**C**) Kα emission of C; (**D**) Kα emission of P; (**E**) Kα emission of F; (**F**) Kα emission of O; (**G**) Kα emission of N; (**H**) Kα emission of Si. Scale bars represent 100 µm.

**Figure 4:**
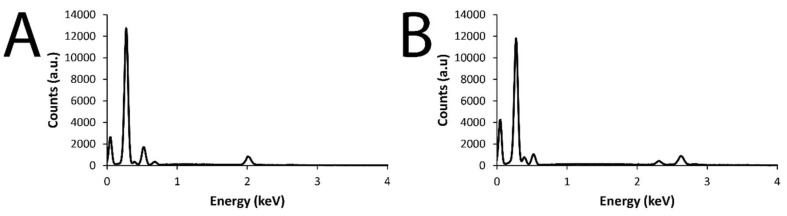
(**A**) EDX data from a DMP-doped PPY film; (**B**) EDX data from an MER-doped PPY film.

The successful deposition of polypyrrole films onto the surface of the ITO electrodes was easily observable by eye (i.e., presence of a black film on the surface of a clear and colorless ITO electrode), and confirmed by scanning electron microscopy (SEM) and energy dispersive X-ray spectroscopy (EDX) data, as shown in Figure 2 (SEM and EDX imaging of DMP-doped films), Figure 3 (SEM and EDX imaging of MER-doped films), and Figure 4 (EDX data for DMP-doped and MER-doped films).

SEM revealed the surfaces of the films to have µm-scale roughness characteristic of electropolymerized PPY, with the features having a broad distribution of sizes, from very small particles of ca. 100–200 nm to much larger 1–20 µm “cauliflower-like” structures, akin to the features reported for films prepared via analogous electropolymerization methodologies in the literature. There were concomitant differences in electrical properties (i.e., impedance/resistance) relative to the surface-area-to-volume ratio of the materials [16,46]. EDX analysis [47] showed elemental signals characteristic of Au (instrumental background, characteristic Kα 2.123 keV; data not shown), Si (glass electrode, characteristic Kα 1.74 keV), and the elements associated with the drug-loaded polymer films: C and N (characteristic of polypyrrole) [48], F, O, and P (characteristic of DMP), and O and S (characteristic of MER).

The EDX maps in Figure 2 and Figure 3 demonstrate the elemental composition of the films to be homogeneous over the surface of the films. Analysis of the EDX data for DMP-doped or MER-doped films (Figure 4A,B, respectively) showed elemental signals characteristic of carbon (Kα at 0.277 keV) and nitrogen (Kα at 0.392 keV) found on polypyrrole. For the DMP-doped films, there were peaks at 0.677 keV, 0.525 keV, and 2.014 keV, characteristic of the Kα of F, O, and P, respectively (Figure 4A). For the MER-doped films, there were peaks at 0.525 keV, 2.308 keV, and 2.622 keV, characteristic of the Kα of O, S, and Cl, respectively (indicative of the hydrochloride salt of MER, Figure 4B).

**Figure 5:**
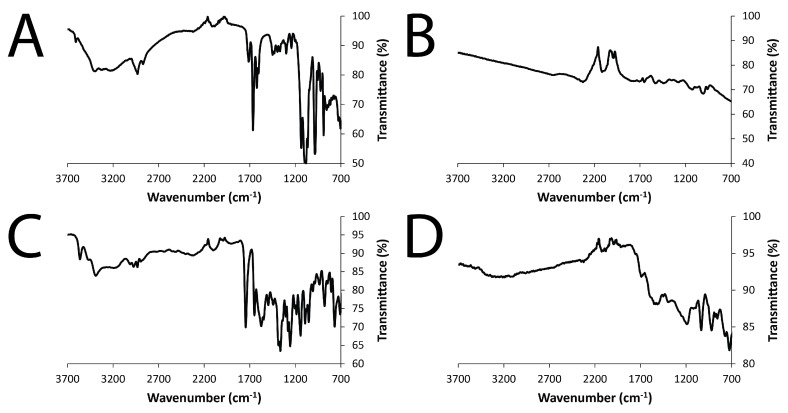
Fourier-transform infrared (FTIR) spectra collected in attenuated total reflection (ATR) mode: (**A**) DMP; (**B**) DMP-doped PPY film; (**C**) MER; (**D**) MER-doped PPY film.

Examination of the films using Fourier-transform infrared (FTIR) spectroscopy in attenuated total reflection (ATR) mode also confirmed the presence of the drugs (DMP and MER) doping the PPY (Figure 5). The FTIR spectra of the PPY films showed absorptions at ca. 1480 cm^−1^ and ca. 1535 cm^−1^, corresponding to the symmetric and asymmetric ring-stretching modes, respectively [49,50,51,52,53]. The FTIR spectra of DMP and DMP-doped PPY films showed absorptions at 989 cm^−1^ and 1197 cm^−1^, corresponding to the characteristic symmetric and asymmetric stretching vibrations of the phosphate groups, while the absorption band at 1641 cm^−1^ corresponded to the C=O stretching vibration of DMP (Figure 5A,B). The FTIR spectra of MER and MER-doped PPY films showed absorptions characteristic of stretching vibrations of the C=O bond in the β-lactam ring of MER, located at 1863 cm^−1^ (Figure 5C,D).

**Figure 6:**
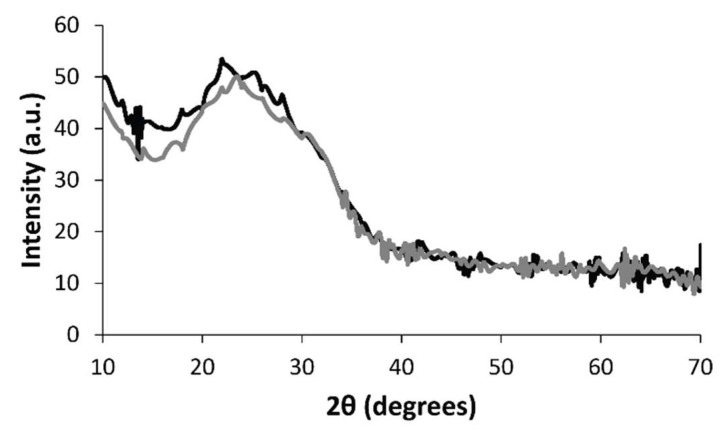
X-ray diffraction (XRD) data. (Black line) DMP-doped PPY film. (Gray line) MER-doped PPY film.

X-ray diffraction (XRD) analysis of the DMP-doped and MER-doped films revealed some interesting structural information confirming the inclusion of the drugs in the films (Figure 6). PPY has a relatively amorphous structure with a broad peak in the region of 2θ = 20–30° in the XRD patterns [54] which is associated with the closest distance of approach of the planar aromatic rings of pyrrole (e.g., face-to-face pyrrole rings) [55]. Interestingly, doping PPY with DMP led to a peak shift to the region of 2θ = 15–28°, confirming that addition of DMP alters the packing of the PPY chains in the film [56]; likewise, doping PPY with MER also led to a peak shift to the region 2θ = 15–38°. Interestingly, some of the crystalline peaks of pure MER (which appear at 12.6°, 16.6°, 18.2°, 19°, 20°, 21.6°, 22.3°, 22.7°, 23.3°, 25.2°, 26.7°, 28.2°, 29°, 30°, 31.7°, 34.5°, 37.7°, 39°, and 44° [57]) can be identified in the XRD pattern (appearing, albeit weakly, at 2θ = 17.9°, 21.9°, 23.7°, 34.5°, and 38.0°). 

**Figure 7:**
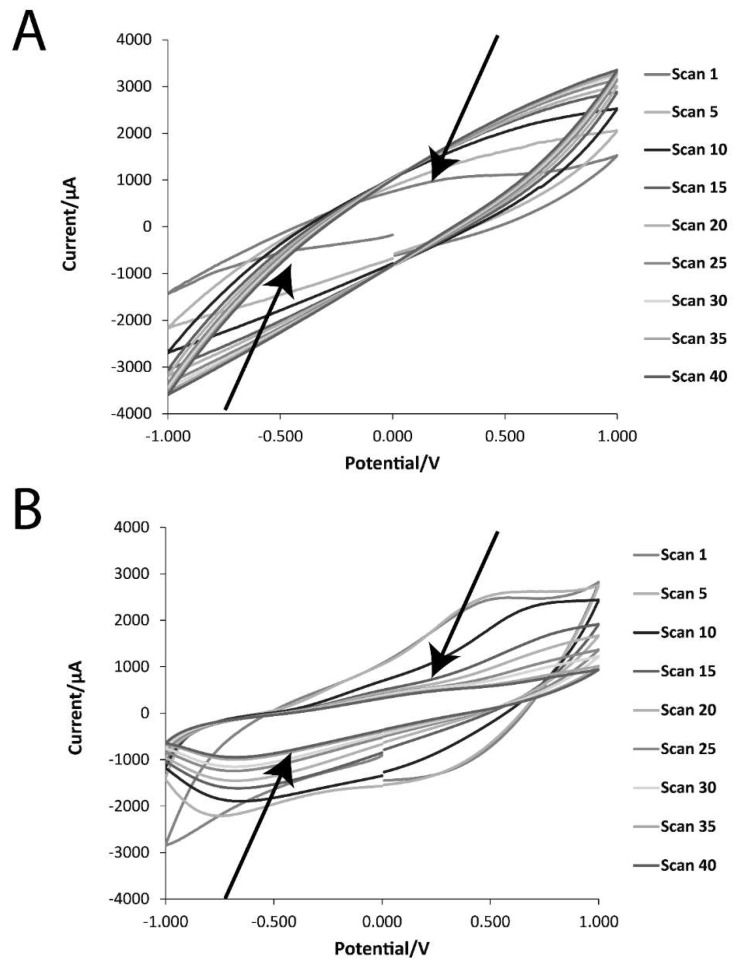
Cyclic voltammetry (CV) data of the films in phosphate-buffered saline (PBS; pH = 7.4) at a scan rate of 50 mV·s^−1^: (**A**) DMP-doped PPY film; (**B**) MER-doped PPY film.

**Figure 8:**
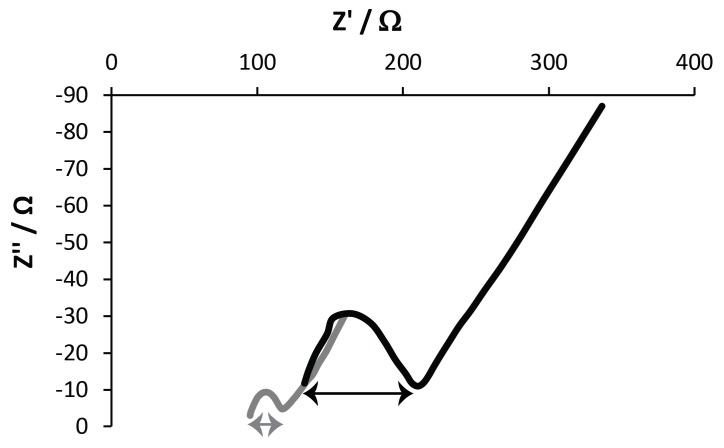
Nyquist plots derived from electrochemical impedance spectroscopy (EIS) data of the films in PBS (pH = 7.4). (Black line) DMP-doped PPY film. (Gray line) MER-doped PPY film.

The electrochemical properties of the DMP-doped PPY and MER-doped PPY films were studied via cyclic voltammetry (CV, Figure 7), and electrochemical impedance spectroscopy (EIS, Figure 8). Cyclic voltammograms of DMP-doped PPY and MER-doped PPY films (1st, 5th, 10th, 15th, 20th, 25th, 30th, 35th, and 40th cycles) are displayed in Figure 7A,B, respectively. As evident from the CV curves, the charge-storage capacities of the films decreased steadily on repeated cycling, and the areas under the curves of the 35th and 40th cycles were almost the same. The currents evolved in the MER-doped PPY were higher than those in the DMP-doped PPY, and the oxidation and reduction peaks were more prominent. Occurrences of reduction and oxidation peaks correspond to the de-doping of the films (i.e., release of drug molecules), and the films were subsequently re-doped by other anions (either the anionic drug, or anions from the PBS buffer: H_2_PO_4_^−^ and HPO_4_^2−^, and Cl^−^).

EIS measurements were conducted to investigate the electron-transfer resistance (R_et_) of the drug-doped PPY films, and the respective Nyquist plots are displayed in Figure 8. All plots have a semi-circular arc in the high-frequency range, followed by a vertical line along the imaginary axis corresponding to a diffusion process. The diameter of the suppressed semicircle gives the value of the electron-transfer resistance (R_et_), which was the most directive and sensitive parameter reflecting the changes at the electrode–solution interfaces, and could be evaluated from the difference in the real part of the impedance between low frequency and high frequency [58]. The R_et_ values were 21.18 Ω and 76 Ω for DMP-doped PPY and MER-doped PPY films, respectively. The R_et_ of MER-doped PPY films was higher than that of DMP-doped PPY films, indicating that the electron transfer was more easily achieved at the DMP-doped PPY film interfaces. The diffusion resistance of DMP-doped PPY films was shorter than that of MER-doped PPY films, indicating a shorter ion-diffusion path length of the [Fe(CN_6_)]^3−/4−^, H_2_PO_4_^−^, HPO_4_^2−^, and Cl^−^ ions into the interior of the film. Importantly, the EIS results are in good agreement with the CV results.

**Figure 9:**
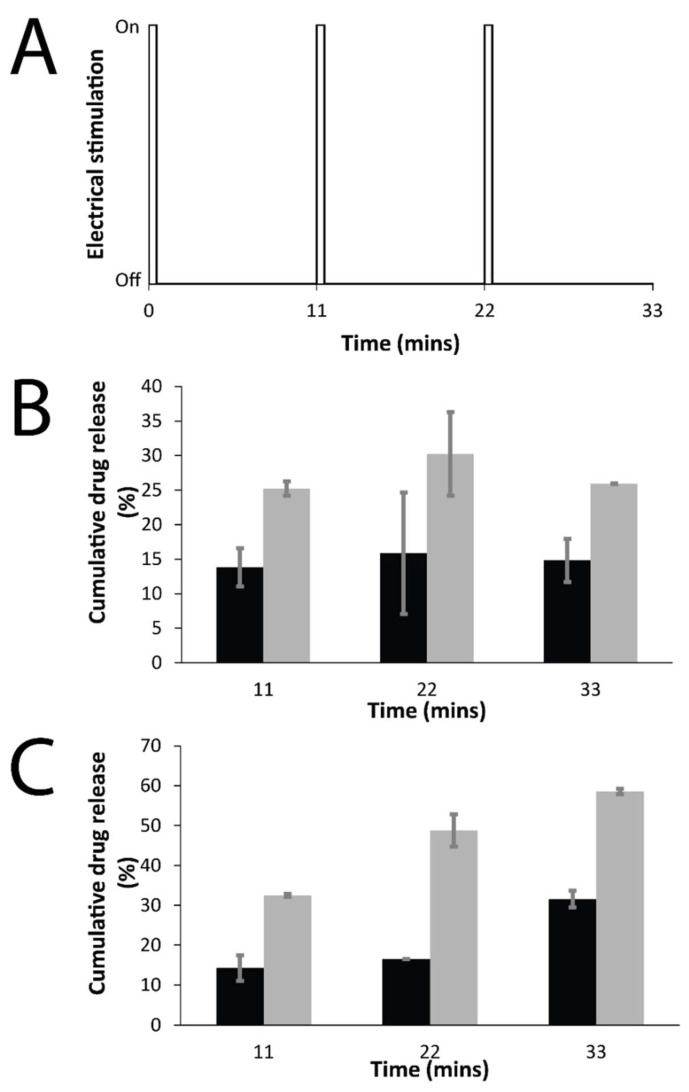
Electrochemically enhanced delivery of drugs from films in PBS (pH = 7.4) as determined by UV spectroscopy: (**A**) Electrical stimulation paradigm: three cycles of 30 s on, 10.5 min off; (**B**) cumulative release of DMP from DMP-doped PPY films, passive release (black bars), electrically stimulated release (gray bars); (**C**) cumulative release of MER from MER-doped PPY films, passive release (black bars), electrically stimulated release (gray bars).

The release of drugs doped into the PPY films was studied using UV spectroscopy (Figure 9), where the release was either passive (i.e., in the absence of an electrochemical stimulus) or electrochemically triggered (i.e., in the presence of one or more rounds of electrochemical stimulation—30 s of stimulation at a reducing potential of 0.6 V, followed by 10.5 min of rest (Figure 9A)). The quantity of the drug in solution was quantified at various time points, and the data are reported as cumulative release as a percentage of the total mass of the drug in the film (films were individually weighed; DMP-doped PPY films contained 12 wt % of DMP, and MER-doped PPY films contained 4 wt % of MER), and compared to passive drug release from unstimulated films every 11 min. 

For both DMP-doped and MER-doped films, the drugs were observed to passively diffuse from the films, as is the norm for drug-loaded polymers. The passive diffusion of DMP from the films was ca. 10–15% of the total DMP content of the films over the course of the experiment, whereas the passive diffusion of MER from the films was ca. 10–30% of the total MER content of the films over the course of the experiment. The amount of drug released from the electrically stimulated films was observed to be higher than that for the passive control samples at each time point measured. For the DMP-doped films (Figure 9B), there was an increase of ca. 10–15% in the amount of drug released at various time points; whereas for the MER-doped films (Figure 9C), there was a an increase of ca. 15–30% in the amount of drug released at various time points after each round of electrical stimulation.

Some physical descriptors (constitutional and electronic) for DMP and MER were calculated using the Molecular Operating Environment (MOE) software (version 2014.0901, Chemical Computing Group Inc., Montreal, QC, Canada). The selected descriptors were the dipole moment, LogP (octanol/water), molecular globularity, number of H-atom donors and acceptors, and molecular flexibility (Table 1). The dipole of DMP was lower than that of MER (1.7033 vs. 9.2305, respectively), as was the number of hydrogen-bond acceptors (8 vs. 9, respectively), hydrogen-bond donors (5 vs. 7, respectively), and flexibility (5.3661 vs. 8.8623, respectively). The globularity of DMP was higher than that of MER (0.1110 vs. 0.0265, respectively), as was the LogP (1.2640 vs. −0.5960, respectively).

## 3. Discussion

Films of PPY loaded with an anionic drug (either DMP or MER, see Figure 1) were deposited onto the surface of ITO-coated glass electrodes via electropolymerization, yielding films where the positive charges on the backbone of the polypyrrole were counterbalanced by the presence of the anionic dopants from the electrolyte during the electropolymerization reaction (in this case, one of the anionic drugs, DMP or MER). PPY films prepared via electropolymerization on flat electrodes typically display cauliflower-like morphologies [16,46]. 

Clearly, surface morphologies and surface-area-to-volume ratios of materials used for drug delivery play an important role in the rate of release of the payloads, and it was observed that mass transport from PPY films with low surface-area-to-volume ratios (e.g., the cauliflower-like morphology) released drugs more slowly than materials with high surface-area-to-volume ratios (e.g., nanowire-like PPY) [16,46,59].

The films were observed to be somewhat imperfect with cracks and inhomogeneities observable (visually or via SEM), and they occasionally delaminated from the underlying ITO electrode. Such problems (i.e., cracks/inhomogeneities and delamination) may be solved using alternative materials for the underlying electrode (e.g., gold, glassy carbon, etc.); through the use of composites, wherein the polymeric dopant forms an interpenetrating network with the conducting polymer binding them together [60,61,62,63], or through the development of heat/solution processable electroactive block copolymers, in which one block is electroactive and the other block is heat/solvent responsive [64,65]. Analysis via spectroscopy (EDX, FTIR, and UV-vis), XRD, and electrochemical techniques (CV and EIS) confirmed the presence of the drugs in the films, and subtle differences in the packing of the PPY chains in the films in the presence of each drug. Interestingly, measurements of the total mass of the drug in the films revealed differences in loading, with DMP-doped PPY films containing 12 wt % of DMP, and MER-doped PPY films containing 4 wt % of MER. The physical descriptors for DMP and MER (Table 1) offered an explanation as to why this was observed: the dipole of DMP was lower than that of MER (1.7033 vs. 9.2305, respectively), as was the number of hydrogen-bond acceptors (8 vs. 9, respectively) and hydrogen-bond donors (5 vs. 7, respectively). Furthermore, the LogP of DMP was higher than that of MER (1.2640 vs. −0.5960, respectively). Consequently, the more hydrophobic drug (DMP) was more readily loaded into the hydrophobic PPY matrix than the hydrophilic MER. 

For both DMP-doped and MER-doped films, the drugs were observed to passively diffuse from the films, as is the norm for drug-loaded polymers. The passive diffusion of DMP from the films was ca. 10–15% of the total DMP content of the films over the course of the experiment, whereas the passive diffusion of MER from the films was ca. 10–30% of the total MER content of the films over the course of the experiment. 

The application of a reducing potential of 0.6 V (for 30 s) to the drug-loaded PPY films resulted in the proactive release of the anionic drug from the films, and rest periods (10.5 min) between the applications of electrical potential offered opportunities for re-doping by other anions (either the anionic drug, or anions from the PBS buffer: H_2_PO_4_^−^ and HPO_4_^2−^, and Cl^−^). We observed the amount of drug released from the electrically stimulated films (including the rest period) to be higher than that for the passive control samples at each time point measured (every 11 min). For the DMP-doped films (Figure 9B), there was an increase of ca. 10–15% in the amount of drug released at various time points; whereas for the MER-doped films (Figure 9C), there was an increase of ca. 15–30% in the amount of drug released at various time points after electrical stimulation. The differences in the in vitro release behavior observed for DMP and MER, despite their close molecular weights, could be attributed to differences in their physical descriptors (such as the dipole moment, total number of hydrogen-bond donors and acceptors present, molecular flexibility, LogP, and molecular globularity), which confirmed MER to be more hydrophilic and polar than DMP, rendering MER easier to release via diffusion from the PPY matrix, and therefore, more responsive to electrical stimuli (Table 1) [66].

The clinically translated examples of electronic interfaces for the body are all currently metal-based. The mechanical properties of these metals are markedly different from the soft tissues in which they are implanted. This mechanical mismatch leads to inflammatory responses and the formation of scar tissue around the electronic interface. A topic of intense current research interest is the development of soft conductive OEM-based coatings for the surface of the metal electrodes, and it is attractive to be able to deliver bioactive substances from the electrode coating (e.g., anti-inflammatories such as DMP). It is also noteworthy that the surgical procedures necessary to implant such devices are complex, and problems associated with microbial infections in the proximity of these devices can potentially be addressed through the delivery of antimicrobials (e.g., MER) from the surface coatings. The release of the clinically relevant drugs (DMP or MER) loaded into the PPY films was observed to be enhanced by the application of an electrochemical stimulus, thereby demonstrating proof of concept that such materials may form a useful conformal coating on the surface of implantable medical devices, potentially diminishing adverse reactions to their implantation in vivo [16,24,60].

## 4. Materials and Methods 

### 4.1. Materials

Unless otherwise stated, all chemicals and consumables were used as received without further purification. Pyrrole (Py, 98% reagent grade), meropenem trihydrate (MER) United States Pharmacopeia (USP) Reference Standard, dexamethasone 21-phosphate disodium salt (DMP), phosphate-buffered saline tablets (PBS, pH 7.4), and indium tin oxide (ITO)-coated glass electrodes were supplied by Sigma-Aldrich. Potassium ferrocyanide trihydrate (>99%) and potassium ferricyanide (>99%) were supplied by Acros Organics (Fisher Scientific, Hampton, NH, USA).

### 4.2. Preparation of Films via Electropolmerization

Indium tin oxide (ITO)-coated glass electrodes with dimensions of 2.5 cm in length and 1 cm in width were cut to size using a diamond pencil. The conductive sides of the glass electrodes were determined with a multimeter (Metrix MX 51, ITT Instruments, Paris, France), and a piece of wire (5 cm, tinned copper, RS Components, Northants, UK) was connected to the conductive side of the glass electrode with copper tape (Diamond Coating Ltd., Halesowen, West Midlands, UK), and wrapped with electrical insulating tape (Advance Tapes, Leicester, UK). Electropolymerizations were performed using a PalmSens EmStat 3+ potentiostat connected to a personal computer and PSTrace 7.4 software (PalmSens, Houten, The Netherlands). A three-electrode system was used with an Ag/AgCl reference electrode (CH Instruments, Inc. Austin, TX, USA), a platinum-mesh counter electrode (Sigma Aldrich, Gillingham, UK; used as a cathode during the electropolymerization), and an ITO-coated glass slide working electrode (used as an anode during the electropolymerization).

The PPY–drug films were deposited onto the ITO anode from solutions containing pyrrole (0.9 M), and either MER or DMP (1 M) in 4 mL of distilled water. Films were deposited onto the ITO working electrode by applying an oxidizing potential of 1.0 V versus the reference electrode for 30 min. After electropolymerization, the films were rinsed with distilled water (ca. 10 mL for ca. 15 s) to remove unreacted monomers and drugs, and were left to dry in air at laboratory temperature (21 °C) for 24 h.

### 4.3. Scanning Electron Microscopy (SEM) Studies

The surfaces of the films were analyzed with scanning electron microscopy (SEM) using a JEOL JSM-7800F SEM (JEOL UK, Welwyn Garden City, UK).

### 4.4. Fourier-Transform Infrared (FTIR) Spectroscopy Studies

Spectra were an average of 16 scans, and were obtained at a resolution of 1 cm^−1^ using an Agilent Technologies Cary 630 FTIR instrument (Agilent Technologies Ltd., Cheadle, UK).

### 4.5. X-ray Diffraction (XRD) Studies

X-ray diffractograms were collected using a Rigaku Smartlab powder diffractometer (Rigaku Ltd., Kent, UK) equipped with a DTex250 one-dimensional (1D) detector, irradiating the films at a wavelength of 0.15418 nm, from Cu Kα radiation. The Cu source was operated at 45 kV and 200 mA, and was fitted with parallel beam optics, with a scan range of 2θ = 10–90°.

### 4.6. Electrochemical Characterization of Films

Cyclic voltammetry (CV) measurements were performed using a PalmSens EmStat 3+ potentiostat connected to a personal computer using the PSTrace 7.4 software, whereas electrochemical impedance spectroscopy (EIS) measurements were performed using an Ivium-n-Stat Multichannel Electrochemical Analyzer. For CV and EIS measurements, a three-electrode system was used with an Ag/AgCl reference electrode (CH Instruments, Inc. Austin, TX, USA), a platinum-mesh counter electrode (Sigma Aldrich, Gillingham, UK), and an ITO-coated glass slide working electrode. The electrodes were in a biomedically relevant buffer (4 mL of phosphate-buffered saline [PBS] at pH 7.4).

For CV measurements, the potential was swept between −1.0 V and +1.0 V vs. the Ag/AgCl electrode at a scan rate of 0.05 Vs^−1^.

For EIS measurements, the PBS also contained [Fe(CN_6_)]^3−/4−^ (5 mmol L^−1^), and measurements were performed with an open-circuit potential of 230 mV, with an amplitude of applied potential perturbation of 10 mV in the frequency range of 0.1–105,000 Hz. The Nyquist plots were obtained to ascertain the electron-transfer resistance (R_et_).

### 4.7. Drug-Delivery Studies

The electrochemically triggered release of MER or DMP from the films was achieved using a PalmSens EmStat 3+ potentiostat connected to a personal computer using the PSTrace 7.4 software (amperometric technique), and a three-electrode system (described above) in a biomedically relevant buffer (4 mL of PBS at pH 7.4). Prior to electrical stimulation, there was a quiet time of 20 s at a potential of 0.1 V, after which the PPY-coated ITO working electrodes were stimulated for 30 s with a reducing potential of 0.6 V. The films were allowed to rest for 10.5 min after each stimulation, during the last 30 s of which 10 µL of the solution was taken for quantification of drug release with UV spectroscopy (at either 242 nm for DMP, or 297 nm for MER) using a Nanodrop 2000c spectrophotometer (Thermo Fisher Scientific, Loughborough, UK). The PBS was not changed between rounds of stimulation, and the data are reported as cumulative release as a percentage of the total mass of the drug in the film (films were individually weighed; DMP-doped PPY films contained 12 wt % of DMP, and MER-doped PPY films contained 4 wt % of MER). These data were compared to the passive drug release from non-stimulated films every 11 min. To determine the total amount of drug in the films, the drug-doped films were stimulated at a reducing potential of 0.6 V for 60 min, and the medium was changed every 10 min. All reported data were normalized relative to the amount of released drug from a 1-mg-drug-doped PPY film with a surface area of 100 mm^2^.

### 4.8. Calculating the Main Physical Descriptors of the Investigated Drugs

Physical descriptors (constitutional and electronic) were calculated. The selected descriptors were the dipole moment, LogP (octanol/water), molecular globularity, number of H-atom donors and acceptors, and the molecular flexibility. The descriptors were calculated using the MOE software version 2014.0901 (Chemical Computing Group Inc., Montreal, QC, Canada), and the builder tool in the same software was used to generate the three-dimensional (3D) structures of the investigated drugs from their isomeric simplified molecular-input line-entry system (SMILES) obtained from The PubChem Project^®^ (National Institutes of Health, Bethesda, MD, USA).

## 5. Conclusions

Electroactive drug-loaded polymeric films represent an effective means of controlling the delivery of various types of drugs, and the development of new on–off therapies from surface coatings with drug-loaded conducting polymers applied to implantable devices. Such coatings have prospects for positive economic and health impacts in the short–medium term (e.g., as coatings for neural electrodes) [35,36], and societal impacts in the long term (e.g., enhanced quality of life). Likewise, the development of biodegradable alternatives to PPY has prospects for health impacts in the long term (e.g., as drug-delivery devices and scaffolds for tissue engineering) [37].

## Figures and Tables

**Table 1:** Physical descriptors of dexamethasone phosphate (DMP) and meropenem (MER).

Drug	Dipole	Number of Hydrogen Bond Acceptors	Number of Hydrogen Bond Donors	Globularity	Flexibility	LogP (octanol/water)	Molecular Weight (Da.)
DMP	1.7033	8	5	0.1110	5.3661	1.2640	472.4460
MER	9.2305	9	7	0.0265	8.8623	−0.5960	437.5140
